# Photograph Based Evaluation of Consumer Expectation on Healthiness, Fullness, and Acceptance of Sandwiches as Convenience Food

**DOI:** 10.3390/foods10051102

**Published:** 2021-05-16

**Authors:** Purificación García-Segovia, Mª Jesús Pagán-Moreno, Amparo Tárrega, Javier Martínez-Monzó

**Affiliations:** 1 Food Technology Department, Universitat Politècnica de València, 46022 Valencia, Spain; jpagan@tal.upv.es (M.J.P.-M.); xmartine@tal.upv.es (J.M.-M.); 2 Instituto de Agroquímica y Tecnología de Alimentos (IATA-CSIC), 46980 Paterna, Spain; atarrega@iata.csic.es

**Keywords:** visual assessment, sandwiches, consumer expectations, acceptance, healthiness

## Abstract

Sandwiches are the most common “casual-food” consumed by all age groups in Spain. Due to the importance of visual appearance to promote unplanned or impulse buying, foodservice and hospitality companies focus on improving the visual impression of their food menus to create an expectation that satisfies both sensory and hedonic consumer experiences. To provide a list of attributes about the visual appearance of sandwiches, 25 students were recruited from a university and were invited to participate in two nominal group technique (NGT) sessions. To understand whether a sandwiches’ appearance can influence the expectation of consumers, 259 participants completed an online survey specially designed from the results of the NGT sessions. Data were analyzed using conjoint, internal preference mapping and cluster analysis; the interaction effect by gender was also studied. The conjoint results indicate that visual perception about the filling (vegetal or pork based) plays the most key role overall in consumer expectation. When consumers choose vegetables as the filling, the consumers’ perceived sandwiches as healthier, but the pork filling was perceived as more attractive and satiating. Interaction effect by gender was observed in filling when females perceived pork filling as less healthy than vegetable. By acceptance, consumers were segmented into three groups. The first cluster (*n* = 80) selected the pork filling. The smaller group (cluster 3, *n* = 36) prioritized the vegetal filling, and the most numerous cluster 2 (*n* = 140) liked sandwiches with multigrain bread. These results may help companies to build tailor-made marketing strategies to satisfy consumer segments.

## 1. Introduction

Food choice is a complex process that influences consumers’ nutrient intake in a food company’s new product development [1,2]. Beyond satisfying physical hunger, people’s food choices are conditioned by previous individual experiences, and culturally shared expectations created on foods. The hedonic response to how a new food is perceived will depend on the divergence between the sensory attributes and the prior expectations, and on other inherent factors from the consumer [3]. These expectations may influence decisions about ingredients, portion size, food type, and nutritional properties. Within the context of sensory analysis and food acceptance research, the cognitive construct of expectations can be applied to both sensory and hedonic experience [4].

The decision to purchase a food item results from two opposite cognitive processes: intuition and rational thinking. Intuitive processes are fast, automatic, and emotional, whereas rational thinking is a slow, effortful, and controlled process [2,5]. Aspects such as food price and health claims can be considered as rational thinking whereas factors such as purchase environment, promotion, packaging, and mood are related to an impulse or intuitive purchase [6,7]. Likewise, Spence et al. [8] suggested that visual appearance promotes unplanned or impulse buying food items, whereas sensory properties prioritize food quality. Previous studies [9,10] investigated the impact of color and color combinations on visual attractiveness in food. These investigations showed evidence that he visual attractiveness of food affects its acceptance. Miao and Mattila [11] studied what food motive (health or indulgence) influence consumers’ impulse buying behavior. Their results suggested that people prioritized emotional needs (“feel good”) versus primary needs (“feel healthy”). Since food choice plays a critical role to have long and healthy lives, it is necessary to understand which factors play a crucial role in decision-making (rational or impulsive) of food [8]. Healthiness and attractiveness are a complex concept that may be gender biased. The effect of gender differences in diet and social media [12] and online cooking [13] has been investigated to provide food recommendations taking into account these differences and to know how gender differences impact in the field of diet, cooking, internet, and food preferences.

An interesting study applying machine learning techniques to predict the preferred recipes using low-level image features and recipe meta-data as predictors was presented by Elsweiler et al. to improve the selection of recipes towards healthier [14,15].

To attract potential customers’ attention to particular food items, the food service and hospitality industries have paid greater attention to improve the visual impression of the food selection, to persuade consumers to buy their products [16]. Visual appearance is a key factor for affective responses toward the food we choose and eat [17]. Using visual stimuli in consumer studies has also increased during the last years [18,19,20,21,22,23,24,25]. Using images of food is easier than manipulating food products in research in the eating domain [26]. Moreover, the visual appearance packaging [4,27,28] may also create expectations in the consumer who may be interested in purchasing the product. In this context, it is important to rely on methodologies that allow precise identification of consumer expectations generated by visual impression.

The nominal group technique (NGT) is a qualitative methodology for data collection that can assess consumer preferences and ranking information [29,30]. In the NGT basic method, the numbers each problem/solution/decision receives are counted, and the solution with the highest (i.e., most favored) total ranking is selected as the final decision [31]. This technique could be an interesting tool for exploring consumers’ expectations of food products.

Sandwiches are the most “casual-foods” consumed by all age groups in Spain [32]. Typically, the chain restaurants specializing in sandwiches offer their products through attractive images to capture consumer attention to drive their food choice. Thus, they were the foods selected for this study where consumer expectations are evaluated through visual appearance of these products.

An extensive literature exists showing relationship between visual appearance and consumer expectations for a particular food [17,33]. The objectives of this work were (i) to obtain a list of attributes about the visual appearance driving the consumer to select a sandwich and (ii) to understand whether the appearance of sandwiches can influence the consumers’ expectation.

## 2. Materials and Methods

### 2.1. Nominal Group Technique (NGT) Procedure

Two NGT sessions, lasting between 1 and 1.5 h each, were held at the Universitat Politècnica de València in January 2016. A total of 25 participants were recruited between Food Science and Technology undergraduate students regarding a consumption profile (consumption of a sandwich at least once a week). All students participated in both sessions. Nominal groups were conducted according to standard procedures like those recommended by Delbecq and Van de Ven [34]. The Special Committee was informed, and this study reviewed, approved, and all participants singed an informed consent before each session.

In the first session, a context was evoked reading a brief at the beginning of the session. After, this sentence was written on blackboard to be present all session. The sentence was: “Imagine that it is lunchtime, you are at your favorite casual-food restaurant and you have to choose a sandwich to lunch”. They were asked, “What could drive your decision to choose a sandwich? What do you expect from a sandwich?” [35]. Then participants worked individually for 5 min to generate written responses (words or short phrases) for those questions. After this period, they shared their answers with the group in a round-robin fashion, and finally they ranked the items that were most relevant to them [29].

The structure to the second session was the same and participants were asked to generate responses to the question: “what is, in your opinion, the most important characteristic in a sandwich?”. Like the previous session, participants answered the question individually and then shared their responses and classified the attributes of the generated list according to their importance.

In both sessions, all responses were discussed with the research team. Similar items were combined when participants, through consensus [31], provided no distinction. Conclusions from these sessions were used to identify attributes, level, and consumer expectations for experimental design.

### 2.2. Experimental Design, Sample Preparation, and Food Image Capture

Attributes and levels of sandwiches were defined based on data analyzed from the NGT discussion. The platform used for online surveys recommended an average duration from 10 to 15 min to respond a questionnaire to reduce the quit rate. A pilot test to check how long it was taking to answer questionnaires with three experimental designs was carried out before launch the survey. In these pilot trial, the three, four, and five characteristics in a sandwich obtained in Q3, were used obtaining 8, 16, and 64 sandwiches combinations. Considering time to answer, an experimental design by four attributes with two levels for each one was planned and a series of 16 sandwiches were tested. The experimental design used, and a preliminary version of the picture of the sandwich’s samples is presented in Table 1.

Sandwiches were prepared in the Nutrition Laboratory at University just before taking the pictures. The caloric value was calculated according to the Spanish Food Composition Database [36].

For each sandwich, a set of photographs was taken using a high-resolution digital camera model Sony α-3000 (Sony Corp., Minato, Tokyo, Japan). Each sandwich was photographed on the same white plate (255 mm diameter). Care was taken to maintain a constant lighting condition and viewing angle in each photograph.

### 2.3. Online Survey: Participants and Experimental Procedure

Two hundred and fifty-nine participants (148 female and 111 male) participated in the study. The participants were randomly recruited using purposive convenience sampling. This non-probability method is perhaps used more often than any other sampling in behavioral science research during preliminary research, or when the goal is to reach a gross estimate of results related to a research subject [37,38,39]. Recruitment was via e-mail in Valencia (Spain), based on specific criteria: interest in participating in the study, consumption of sandwich at least once a week, to be omnivorous, without food allergies or intolerances. At the recruitment stage, no information about the specific aim of the study was provided.

The electronic questionnaire implemented for this task was designed in RedJade^®^ Online Survey Tool (Redjade Sensory Solutions, LLC, Martinez, CA, USA) including questions about consumer profiles (gender, age, and food frequency sandwiches intake). To introduce test consumers who were asked to imagine that they were going to the snack bar to buy a sandwich. After this introduction, respondents were asked to value each of the 16 sandwiches pictures, which were presented in a randomized order. To measure consumer’s expectations, a 9-point hedonic scale was used. The link to the form was sent to all participants via e-mail. The online questionnaire was available for 1 week.

### 2.4. Data Analysis

To identify which attributes and levels have the most influence in choice, purchase, and acceptance on consumers’ expectations, conjoint analysis is one of the most widely used methodologies [40,41]. The technique can identify the combination of attributes that utilize the consumer and the relative importance (RI) of predefined attributes in total utility. Conjoint and Cluster Analyses, using XLSTAT Sensory 2021.1.1 (Addinsoft, New York, NY, USA), analyzed responses from online questionnaires [42].

Conjoint measurement was run to evaluate the joint effect of the independent variables (attributes and level of sandwiches) on the ordering of the dependent variables (consumers’ expectations). Similarly, Two-way ANOVA was made to assess gender interaction with sandwiches’ attributes.

An internal preference mapping was performed for consumer acceptance evaluated to segment the consumers into groups of similar criteria, data from the other variables measured were introduced in PCA as supplementary variables. In a second stage, a ‘k-means’ clustering followed by an agglomerative hierarchical clustering (AHC) was used to identify distinct patterns in the responses. If required, conjoint analysis was conducted to each cluster to test if there is a significant difference in each consumers’ expectations between attribute-level combinations.

## 3. Results and Discussion

### 3.1. Nominal Group Technique (NGT)

The aim in the first session with NGT was to select dependent variables (expectations when consumers getting a sandwich) to prepare a questionnaire online in the next survey step. Responses to question 1 (What drove your decision to choose a sandwich?) and 2 (What do you expect from a sandwich?) from the NGT are listed and ranked as seen in Table 2.

Comparing results of NGT for Q1 and Q2, “expectations when people get a sandwich” vs. “driven decision to choose a sandwich”, respondents from both questions mentioned same items: “Attractive/seems good” (translated from “apetecible” in Spanish), “to be healthy”, “fullness/feel full”, and “acceptance/willing to eat” (defined in Spanish as “que me guste”). The most important parameters for all participants, when they select a sandwich, were related to the aspect (healthy, attractiveness, and desirable), and fullness. According to the NGT methodology, participants and the research team discussed the answers and through consensus and selected the top four items as dependent variables to include as questions in the online survey. Using information gathered in the second NGT session, attributes and levels of sandwiches were defined based on responses for question 3, “What’s the most important characteristic in a sandwich?” (Table 3). The investigative team discussed and arrived at a consensus with NGT participants to select the key attributes/levels and define the experimental design.

Table 1 shows the experimental design used where attributes and levels were defined as: (1) sandwich filling: vegetable or pork based; (2) kind of bread: multigrain or tomato; (3) shape of bread: loaf or round; (4) sandwich preparation: toasted or fresh.

Although the sandwiches contained different ingredients, they were prepared to get the same final weight. Table 4 shows the ingredients and weight of each one of the different sandwiches. The average weight of each was 215 ± 5 g for vegetable and 220 ± 4 g in pork sandwiches.

### 3.2. Online Survey

A total of 256 men and women participated in the online survey, filling all questions. Their ages varied from 20 to 65, where 46.1% had ≤25, 35.6% were 26–45, and 27.4% were 46–65. Responses to questions about consumer’s profile (gender, age, food frequency sandwiches intake, and willing to pay) are shown in Table 5.

Of the data collected from the 265 respondents’ online questionnaire, nine data responses were unusable for analysis because the participants failed to complete all questions.

Data were analyzed, individually generating a utility value of each level and the utility importance value of each attribute in each consumer’s expectation (Table 6).

Utility estimate referred numerical scores that measure how much each feature influences the customer’s decision to make that choice. Importance values aggregate into one set consumer preferences of attribute-level utilities. Analyzing the results showed in Table 6 importance values vary for each consumer’s expectation. To “fullness”, the sequence of the highest positive utility values was in decreasing order: filling, bread shape, sandwich preparation, and type of bread. For healthy perception, the highest positive sequence was filling, kind of bread, sandwich preparation, and the shape of bread. For the attractiveness, filling, sandwich preparation, shape, and kind of bread was the positive sequence defined by respondents. Filling and kind of bread were the most valued acceptance attributes.

For all consumers’ conjoint expectation analysis, results indicate that filling (pork or vegetable) was the most valued attribute that affects consumer preferences of sandwiches. Filling, as the key attribute, has the highest utility value for “pork filling” in the consumer’s expectations “fullness”, “attractiveness”, and “acceptance” but it was a negative utility when the “healthy” perception was evaluated.

Consumers chose vegetables for the filling, which indicates that they perceived these as healthier, but pork filling was identified as more attractive and satiating. An explanation for this perception of healthiness of vegetables could be that this product has a strong health image versus pork, conveyed to the consumers like the concept low in calories. A study from Rebollar et al. [43] shows a clear relationship between the attributes of low-calories and healthy. The Mediterranean diet (MD) was defined as a daily intake of plant foods and moderate amounts of meat; its recommendations have had a substantial impact as the best “healthy eating model”. Thus, consumer perception of sandwiches can be related to consumer’s experience and learning about MD, suggesting this can improve acceptance ratings in choice behavior [44,45,46].

### 3.3. Internal Preference Mapping and Cluster Analysis

**Figure 1:**
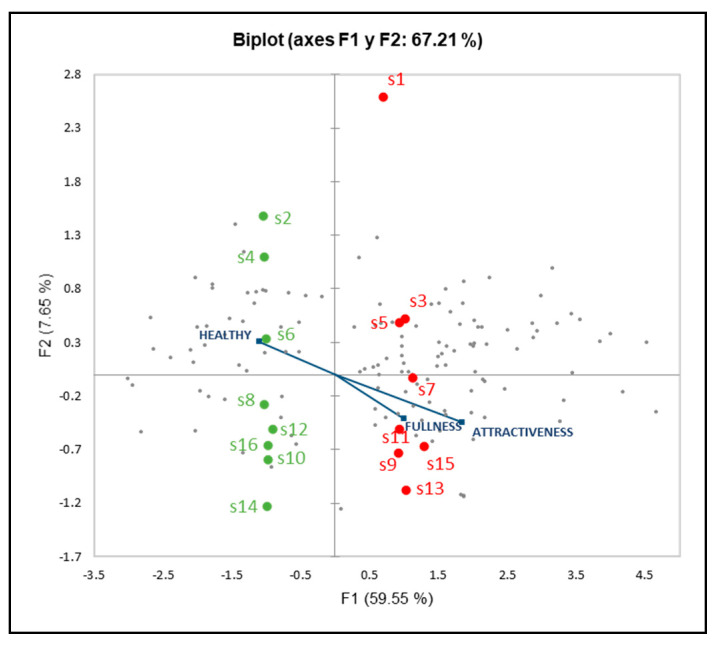
Consumer internal preference map obtained from acceptance ratings and projection of fullness, healthy, and attractiveness as supplementary variables.

An internal preference map was obtained from principal components analysis (PCA) of the four consumer’s expectation data (Figure 1). The two principal components explained 67.21% of the variance for “acceptance”. The dispersion of consumers on the right of the graph indicated some common acceptance pattern between consumers on the F1 but some differences at the same time on the F2. In the Figure 1, F1 separates the “vegetable filling” (S2, S4, S6, S8, S10, S12, S14, and S16) from the “pork filling” (S1, S3, S5, S7, S9, S11, S13, and S15) sandwiches, whereas F2 separates multigrain versus tomato bread. S1 presented a high positive contribution on F2, different than others probably because it is recognized as the most of the most classic and demanded sandwich at Spanish lunch. S1 opposed to the location of S13, showed that even if these two sandwiches had a high consumer’s acceptability, they were not chosen by the same consumers, those who preferred S1(pork-multigrain-loaf-fresh) did not choose S13 (pork-tomato-round-fresh).

Projection of the other consumer’s expectation on IPM allowed to observe the relation with acceptability. Sandwiches with pork filling generated a greater expectation of “fullness”. Vegetal filling sandwiches were observed as a negative value on the first component, with S2 and S4 were the least “fullness” values. According to Fiszman et al. [47], the pork meat could be associated with a harder texture and anticipated to the consumers a prolonged oral processing and oral exposure, contributing to “fullness” perception in pork sandwiches.

To study segmentation observed in PCA, k-means clustering followed by AHC was used to identify distinct patterns.

**Figure 2:**
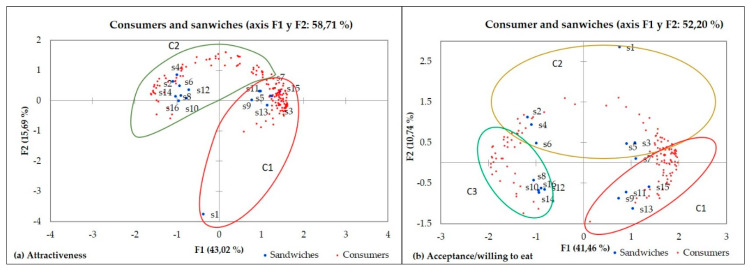
Clusters of respondents for (**a**) attractiveness; (**b**) acceptance.

Two clusters with different attractiveness patterns were obtained (Figure 2a). Cluster 1 (*n* = 86) prioritizes “pork filling” as the key attribute in attractiveness. Cluster 2 (*n* = 170) showed a marked preference for “vegetal filling”, and values positively “toasted bread.” Both consider the kind of bread (multigrain or tomato flavor) as a less important motivating preference by attractiveness.

For acceptance, consumers were segmented into three groups (Figure 2b): the first cluster (*n* = 80) selects pork filling. The smaller group (cluster 3, *n* = 36) prioritize vegetal filling, and the most numerous cluster 2 (*n* = 140) liked sandwiches with multigrain bread. Previous studies of consumer’s perceptions of bread [48,49,50,51] or market trends [52] found higher health-related perceptions of whole grain and high-fiber grain bread. This could explain the high consumer segment with preference for multigrain bread sandwiches in our study.

The results of the ANOVA test for each consumer’s expectation (or their clusters) are presented in Table 7, confirming a significant difference in the attributes for “fullness” and “healthy” and between clusters for “attractiveness” and “acceptance”. In all cases, “filling” was the major contributing attribute.

### 3.4. Gender Effects

Two-way ANOVA revealed a statistically significant effect for gender in “fullness” and “acceptance” expectations for sandwiches (Table 8). Females expect sandwiches to make them more than males (F = 7.33 (0.04) and M = 6.52 (0.05), respectively). The same effect was observed in acceptance perception, where women rated significantly higher on the hedonic scale. Male and female subjects did not significantly differ in their expectations in the “healthy” or “attractiveness” of sandwiches (*p >* 0.05).

There were no significant interaction effects by gender for kind of bread, shape of bread, or sandwich preparation. Interaction effect by gender was only observed in filling for healthy, attractiveness, and acceptance/willing to eat.

Since 1990 some authors have studied the role of gender on food choice [53,54,55,56]. Their results suggest that some gender differences might exist concerning total caloric consumption and preferences for certain types of food. However, various authors defined how visual attributes of the product such as color, shape, pattern, and texture could be often associated with gender-based stereotypes about food [57,58,59,60]. Similar to the results seen by the previous authors, the results of the present study (see Table 6) showed that the pork filling was perceived as less healthy than vegetable; this difference was most evident for women. Although, men did not perceive vegetable sandwiches as healthier, or pork based as unhealthier. In contrast, men referred to more differences between vegetable and pork filling in attractive perception.

Related to acceptance or willing to eat differed between women’s and men’s expectations (*p* < 0.0001), women preferred vegetal filling (Table 6).

## 4. Conclusions

The investigation focused on evaluating sandwiches based on visual perception because in the moment of choice, the consumer does not have access to the product. Notwithstanding the limitations, this study could be considered an exploratory study to understand how individuals choose sandwiches in “casual-food” bar by showing the importance of the visual appearance in these choices. These results may help foodservice companies to build tailor-made menu strategies to satisfy different consumer segments. These results contribute to show the relevance of food images in creating consumers’ expectations on satisfaction, attractiveness, or healthiness, which could affect their product acceptance.

## 5. Limitations

Like other studies, this research did not include a tasting phase. This phase could be interesting to confirm the expectations generated by the photograph evaluation of the sandwiches, with the “fullness” attribute. On the other hand, the concepts “healthiness”, “attractiveness”, or “fullness” are complex, difficult to measure, and may be a gender bias. Future studies should be addressed to measure the different concept of healthiness, fulness, and attractiveness in the participants to identify how visual cues affects food choice. The visual evaluation of the pictures could be affected because not all ingredients were seen. Authors are designing a new experience whereby participants will be provided the information about sandwiches ingredients.

## Figures and Tables

**Table 1:** Description of the sandwiches evaluated by consumers following the experimental design.

Sandwich	Filling	Kind of Bread	Shape of Bread	Making Sandwich	Picture
1	Pork	Multigrain	Loaf	Fresh	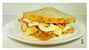
2	Vegetal	Multigrain	Loaf	Fresh	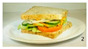
3	Pork	Multigrain	Loaf	Toasted	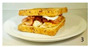
4	Vegetal	Multigrain	Loaf	Toasted	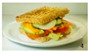
5	Pork	Multigrain	Round	Fresh	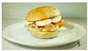
6	Vegetal	Multigrain	Round	Fresh	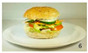
7	Pork	Multigrain	Round	Toasted	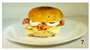
8	Vegetal	Multigrain	Round	Toasted	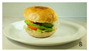
9	Pork	Tomato	Loaf	Fresh	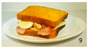
10	Vegetal	Tomato	Loaf	Fresh	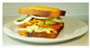
11	Pork	Tomato	Loaf	Toasted	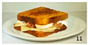
12	Vegetal	Tomato	Loaf	Toasted	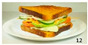
13	Pork	Tomato	Round	Fresh	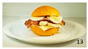
14	Vegetal	Tomato	Round	Fresh	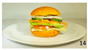
15	Pork	Tomato	Round	Toasted	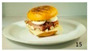
16	Vegetal	Tomato	Round	Toasted	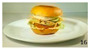

**Table 2:** Nominal group technique: Top 10 responses to the questions Q1 and Q2

Q1. What Drove Your Decision to Choose a Sandwich?		Q2. What Do You Expect from a Sandwich?	
Responses	Total Votes	Responses	Total Votes
Attractiveness	23	It seems/taste good	25
Healthy aspect	21	Fullness/be satisfied	22
Desire to eat	21	Healthy	20
Fullness/be satisfied	20	Desire to eat	18
Caloric value	17	Freshly made	15
Price	15	Adjusted price/quality	14
Succulent	10	Easy to eat	10
Easy to eat	9	Can eat with fingers	8
Can eat with fingers	6	Crisp, well-baked, well-filling	7
To be hungry	5	Convenience packaged	5

**Table 3:** Responses to the question Q3.

Q3. What’s the Most Important Sandwich Characteristic?		
Responses		Total Votes
Attributes	Level	
Filling		25
	Pork	15
	Veggie	10
Sandwich preparation		18
	Fresh	9
	Toast	9
Bread shape (easy to eat)		17
	Round	10
	Loaf	7
Kind of bread		11
	High fiber	6
	Flavored	5
Price (€)		8
	<2	1
	2–2.5	3
	2.5–3	3
	>3	1
Size		5
	Normal	3
	Big	2
Not staining filling		4
Flavor		3
	Spice	2
	Dairy (cheese)	1

**Table 4:** List of ingredients and weight of designed sandwiches.

Sandwich	Ingredients	Grams
Pork	Bread	50
	Pork loin	75
	Bacon	25
	Egg	25
	Cheese	20
	Fried onion	25
	Total	220
Vegetal	Bread	50
	Lettuce	10
	Tomato	40
	Avocado	50
	Cucumber	25
	Carrot	25
	Fresh onion	15
	Total	215

**Table 5:** Consumer’s profile (*n* = 256).

	All	Female	Male
	(*n* = 256)%	(*n* = 145)%	(*n* = 111)%
		56.6	43.4
Age (years)			
<25	46.1	49.7	41.4
26–35	16.8	16.6	17.1
36–45	18.8	17.2	20.7
46–55	12.9	11.0	15.3
55–65	5.5	5.5	5.4
>65	0.0	0.0	0.0
Frequency of consumption (%)			
Never	0.0	0.0	0.0
Occasionally	9.0	9.0	9.0
<4	25.8	29.0	21.6
Between 4–8	31.6	31.0	32.4
>8	33.6	31.0	36.9
Willing to pay (€)			
<2	10.9	9.7	12.6
2–2.5	38.7	40.7	36.0
2.5–3	40.2	37.9	43.2
>3	10.2	11.7	8.1

**Table 6:** Conjoint analysis results for each consumers’ expectations between attributes-level combinations.

			All Consumers (*n* = 256)		Female Group (*n* = 146)		Male Group (*n* = 110)	
Consumer’s Expectations	Attributes	Levels	Utility Estimate	Importance Values	Utility Estimate	Importance Values	Utility Estimate	Importance Values
Fullness	Filling	Pork	0.963	77.3	−0.024	23.4	0.032	23.4
		Vegetal	−0.963		0.024		−0.032	
	Kind of bread	Multigrain	−0.048	3.9	−0.036	35.2	0.048	35.2
		Tomato	0.048		0.036		−0.048	
	Shape of bread	Loaf	−0.17	13.6	−0.035	33.6	0.046	33.6
		Round	0.17		0.035		−0.046	
	Sandwich preparation	Fresh	−0.065	5.2	0.008	7.8	−0.011	7.8
		Toasted	0.065		−0.008		0.011	
Healthy	Filling	Pork	−1.777	68.2	−0.113	81.8	0.150	81.8
		Vegetal	1.777		0.113		−0.150	
	Kind of bread	Multigrain	0.336	12.9	0.001	0.6	−0.001	0.6
		Tomato	−0.336		−0.001		0.001	
	Shape of bread	Loaf	0.213	8.2	−0.014	10.1	0.019	10.1
		Round	−0.213		0.014		−0.019	
	Sandwich preparation	Fresh	0.278	10.7	−0.010	7.6	0.014	7.6
		Toasted	−0.278		0.010		−0.014	
Attractiveness	Filling	Pork	0.463	57.0	−0.115	76.4	0.153	76.4
		Vegetal	−0.463		0.115		−0.153	
	Kind of bread	Multigrain	−0.037	4.6	−0.019	12.4	0.025	12.4
		Tomato	0.037		0.019		−0.025	
	Shape of bread	Loaf	−0.122	15.0	0.013	8.4	−0.017	8.4
		Round	0.122		−0.013		0.017	
	Sandwich preparation	Fresh	−0.191	23.5	0.004	2.7	−0.005	2.7
		Toasted	0.191		−0.004		0.005	
Acceptance	Filling	Pork	0.393	70.4	−0.102	60.4	0.135	60.4
		Vegetal	−0.393		0.102		−0.135	
	Kind of bread	Multigrain	0.116	20.8	−0.043	25.7	−0.057	25.7
		Tomato	−0.116		0.043		0.057	
	Shape of bread	Loaf	0.029	5.2	0.014	8.5	−0.019	8.5
		Round	−0.029		−0.014		0.019	
	Sandwich preparation	Fresh	−0.02	3.6	0.009	5.4	−0.012	5.4
		Toasted	0.02		−0.009		0.012	

Note: Grey color marks the Importance Value most relevant in each consumer expectations.

**Table 7:** ANOVA to test significant differences between attributes-level combinations in the consumer’ expectations.

Consumer’s Expectations	Model		Error			
	Mean Squares	df	Mean Squares	df	F	Pr > F
Fullness (*n* = 256)	986.7163	4	3.9198	4091	251.7290	<0.0001
Healthy (*n* = 256)	3474.4797	4	3.7065	4091	937.3898	<0.0001
Attractiveness (*n* = 256)	273.6882	4	5.7710	4091	47.4248	<0.0001
Cluster 1 (*n* = 86)	1404.9397	4	4.0054	1371	350.7595	<0.0001
Cluster 2 (*n* = 170)	75.3610	4	4.2681	2715	17.6569	<0.0001
Acceptance (*n* = 256)	173.0398	4	4.9937	4091	34.6518	<0.0001
Cluster 1 (*n* = 80)	1013.0570	4	4.1198	1275	245.8992	<0.0001
Cluster 2 (*n* = 140)	15.7353	4	2.6716	2235	5.8899	<0.0000
Cluster 3 (*n* = 36)	414.0191	4	2.8646	571	144.5306	<0.0001

**Table 8:** F-test in two-way ANOVA for gender.

		Fullness	Healthy	Attractiveness	Acceptance
	R^2^	0.232	0.481	0.048	0.039
	F	137.031	420.230	22.800	18.603
	Pr > F	<0.0001	<0.0001	<0.0001	<0.0001
Gender	F	178.036	0.004	2.028	14.354
	Pr > F	<0.0001	0.950	0.154	0.000
Gender × Filling	F	0.845	18.898	12.512	11.329
	Pr > F	0.358	<0.0001	0.000	0.001
Gender × Kind of bread	F	1.920	0.001	0.331	2.042
	Pr > F	0.166	0.976	0.565	0.153
Gender × Shape of bread	F	1.743	0.289	0.151	0.222
	Pr > F	0.187	0.591	0.697	0.637
Gender × Sandwich preparation	F	0.094	0.161	0.016	0.091
	Pr > F	0.759	0.688	0.900	0.763

Note: Grey color marks significant interaction effects.
